# CK1δ restrains lipin-1 induction, lipid droplet formation and cell proliferation under hypoxia by reducing HIF-1α/ARNT complex formation

**DOI:** 10.1016/j.cellsig.2015.02.017

**Published:** 2015-06

**Authors:** Maria Kourti, Georgia Ikonomou, Nikolaos-Nikiforos Giakoumakis, Maria Anna Rapsomaniki, Ulf Landegren, Symeon Siniossoglou, Zoi Lygerou, George Simos, Ilias Mylonis

**Affiliations:** a Laboratory of Biochemistry, Faculty of Medicine, University of Thessaly, Larissa, Greece; b Laboratory of Biology, School of Medicine, University of Patras, Rio, Patras, Greece; c Department of Immunology, Genetics and Pathology, SciLifeLab, Uppsala University, Uppsala, Sweden; d Cambridge Institute for Medical Research, University of Cambridge, Wellcome Trust/Medical Research Council Building, Hills Road, Cambridge CB2 0XY, United Kingdom

**Keywords:** HIF, hypoxia-inducible factor, ARNT, aryl hydrocarbon receptor nuclear translocator, CK1, casein kinase 1, hBSMC, human bronchial smooth muscle cells, PLA, proximity ligation assay, FRAP, fluorescence recovery after photobleaching, Hypoxia, Lipid metabolism, CK1δ, HIF-1, Lipin1, Lipid droplets

## Abstract

Proliferation of cells under hypoxia is facilitated by metabolic adaptation, mediated by the transcriptional activator Hypoxia Inducible Factor-1 (HIF-1). HIF-1α, the inducible subunit of HIF-1 is regulated by oxygen as well as by oxygen-independent mechanisms involving phosphorylation. We have previously shown that CK1δ phosphorylates HIF-1α in its N-terminus and reduces its affinity for its heterodimerization partner ARNT. To investigate the importance of this mechanism for cell proliferation under hypoxia, we visually monitored HIF-1α interactions within the cell nucleus using the *in situ* proximity ligation assay (PLA) and fluorescence recovery after photobleaching (FRAP). Both methods show that CK1δ-dependent modification of HIF-1α impairs the formation of a chromatin binding HIF-1 complex. This is confirmed by analyzing expression of lipin-1, a direct target of HIF-1 that mediates hypoxic neutral lipid accumulation. Inhibition of CK1δ increases lipid droplet formation and proliferation of both cancer and normal cells specifically under hypoxia and in an HIF-1α- and lipin-1-dependent manner. These data reveal a novel role for CK1δ in regulating lipid metabolism and, through it, cell adaptation to low oxygen conditions.

## Highlights

• CK1δ impairs HIF-1 complex formation in intact and living cells.
• CK1δ inhibits lipin-1 induction and lipid droplet formation under hypoxia.
• Inhibition of CK1δ stimulates cell proliferation under hypoxia.

## 1 Introduction

Oxygen deprivation of cells and tissues (hypoxia) causes a dramatic alteration in gene expression and characterizes major pathological processes like ischemia and cancer. The response to hypoxia is mainly mediated by the hypoxia-inducible factors (HIFs) that control the expression of genes involved in metabolic reprogramming as well as angiogenesis, cellular proliferation and survival under low oxygen conditions. HIFs are therefore essential for adaptation to hypoxia that allows cancer cell proliferation in the hypoxic tumor microenvironment or survival of normal cells in ischemic tissue [1,2]. A major part of this adaptation involves HIF-1-mediated stimulation of anaerobic carbohydrate metabolism and repression of oxidative phosphorylation [3]. In addition, HIF-1 is implicated in lipid metabolism by supporting fatty acid synthesis via glutamine metabolism [4], decreasing β-oxidation of fatty acids [5], and increasing lipin-1-dependent neutral lipid synthesis and lipid droplet formation [6].

HIFs act as heterodimers and consist of the regulatory HIFα subunits and the constitutively expressed HIFβ (or aryl hydrocarbon receptor nuclear translocator; ARNT) subunit. Under normal oxygen levels, HIF-1α is modified by prolyl hydroxylases (PHDs), polyubiquitinated and degraded by the proteasome [7]. Under low oxygen tension, hydroxylation is inhibited, and HIF-1α is stabilized and translocated into the nucleus where it interacts with ARNT to form an active DNA-binding heterodimer that associates with hypoxia-response elements (HREs) and activates the transcription of target genes.

HIF-1α expression and transcriptional activity are additionally regulated by oxygen independent mechanisms involving the NF-κB, PI3-K, MAPK and STAT3 pathways [8,9] or interactions with many other proteins including HSP90, RACK1 and MgcRacGAP [10–13]. A key aspect of HIF-1α regulation involves its post-translational modification by protein kinases. Phosphorylation can regulate HIF-1α protein stability, as reported for modifications introduced by kinases GSK3, PLK3 and CDK1 [14–16], or it can affect HIF-1 transcriptional activity. In the latter case, phosphorylation by ERK1/2 at Ser641/643 impairs CRM1-mediated nuclear export of HIF-1α, thereby increasing its nuclear accumulation and activity [17,18]. In addition, we have recently shown that casein kinase 1δ (CK1δ) targets Ser247 at the PAS B domain of HIF-1α, causing reduction of its *in vitro* affinity for ARNT and inhibition of its transcriptional activity [19].

CK1δ is a member of the CK1 protein kinase family that is composed of seven distinct mammalian isoforms (α, β, γ1, γ2, γ3, δ and ε) and their splice variants. Although most of these isoforms are ubiquitously expressed and involved in diverse cellular functions such as cell cycle progression, DNA damage response and circadian rhythms, their regulation is complex and poorly understood [20].

In this work, we investigate the effects of CK1δ-mediated phosphorylation on HIF-1 function in intact and living cells and analyze its involvement in the metabolic reprogramming and proliferation of cells under hypoxia. Our data demonstrate a novel role of CK1δ in limiting lipid biosynthesis and cell proliferation under hypoxia by inhibiting full activation of the HIF-1/lipin-1 axis.

## 2 Materials and methods

### 2.1 Plasmids and antibodies

Cloning of HIF-1α1-347 (N-terminal fragment) into pBS-SK(+) and HIF-1α348-826 (ΔΝ) into pGEX-4T-1 was described previously [21]. The corresponding cDNA inserts were inserted as BamHI fragment into the pEGFP-C1 vector. pEGFP-HIF-1αS247A and pEGFP-HIF-1αS247D plasmids were previously described [19]. pCDNA3.1-CK1δ and pCDNA3.1-CK1δ-K38M [22,23] were kindly provided by Uwe Knippschild (Centre of Surgery, University of Ulm, Germany). Antibodies used included: affinity purified rabbit polyclonal antibodies against HIF-1α [24], lipin-1 and lipin-2 [25], mouse monoclonal antibody against ARNT (BD Biosciences), goat polyclonal antibody against GFP (SICGEN) and rabbit polyclonal antibodies against actin, tubulin (Cell Signaling) or CK1δ (Santa Cruz Biotechnology).

### 2.2 Cell culture, transfection, reporter gene assays and chromatin immunoprecipitation

Cells were cultured in DMEM, for HeLa and Huh7 cells, or DMEM F-12, for human bronchial smooth muscle (hBSM) cells (Lonza), containing 10% FCS and 100 U/ml penicillin/streptomycin (Biochrom). Cells were grown in a 37 °C incubator with 5% CO_2_. For hypoxic treatment, cells were exposed for 4–24 h to 1% O_2_, 94% N_2_ and 5% CO_2_ in an IN VIVO_2_ hypoxia workstation (Baker Ruskinn). When required, cells were treated for 4–24 h with CK1δ inhibitor D4476 (10 μΜ, Cayman Chemical) or kaempferol (50–100 μΜ, Sigma) using a 10 mM stock solution in dimethyl sulfoxide (Applichem). Transient transfections, reporter gene assays and chromatin immunoprecipitation were performed as previously described [6].

### 2.3 siRNA-mediated silencing

HeLa cells were incubated in serum-free DMEM for 4 h with siRNA (10 nM) against HIF-1α (Qiagen) or *Lpin1* [6] in the presence of Lipofectamine™ RNAiMAX (Invitrogen). AllStars siRNA (Qiagen) was used as negative control.

### 2.4 Western blot and fluorescence microscopy

Immunoblotting and immunofluorescence microscopy were carried out as previously described [6]. Western blot images were taken using an Uvitec Cambridge Chemiluminescence Imaging System with the help of Alliance Software (ver. 16.06) and quantified by Uviband Software (ver. 15.03) provided with the instrument (Uvitec Cambridge). To visualize lipid droplets, cells were stained with Nile Red (Sigma; 0.1 μg/ml in PBS) for 15 min, washed with PBS and mounted on slides. Quantification of the surface covered by Nile Red fluorescence was performed with the public domain software for image analysis ‘ImageJ’ and expressed as pixels/cell [26].

### 2.5 Cell proliferation assay

HeLa or hBSM cells (1.000–2.000 cells/well) were seeded into 96-well plates and incubated for 24 h before being treated with 10 μΜ D4476 or with DMSO as solvent control for the indicated periods under normoxic or hypoxic conditions. At the end of the incubation period, cell proliferation was determined using the “CellTiter 96 Aqueous One Solution Cell Proliferation Assay” kit (Promega). Values were normalized by control experiments in the absence of cells in 96-well plates supplied with culture medium and DMSO or D4476 alone.

### 2.6 *In situ* proximity ligation assay

The *in situ* proximity ligation assay (PLA) allows the visualization and subcellular localization of protein–protein interactions in individual fixed cultured cells, using secondary antibodies with attached oligonucleotides. When a pair of antibodies binds in proximity, the attached oligonucleotides can guide the creation of a DNA circle by ligation. This circle then templates a local rolling circle amplification reaction, whose product is easily detectable by FISH [27]. The HIF-1α interaction with ARNT under hypoxia was monitored in HeLa cells grown on slides. After appropriate incubation, cells were fixed with 3% formaldehyde in PBS for 5 min, permeabilized with PBS/Triton 1% for 15 min at 4 °C, incubated with anti-HIF-1α and/or anti-ARNT antibodies for 16 h at 4 °C and processed using the Duolink II Fluorescence Kit (Olink Bioscience). Slides were counterstained with DAPI (100 μg/ml) before mounting. Images of *in situ* PLA experiments were taken in a Zeiss Axioplan fluorescence microscope using an AxioCam MRm CCD sensor and 40 × objective with filters for DAPI, FITC and Cy3. PLA signals were digitally quantified using the ITCN tool of public domain software for image analysis ImageJ [28].

### 2.7 Fluorescence recovery after photobleaching (FRAP) experiments and data analysis

HeLa cells were plated on 35-mm high glass-bottom Ibidi μ-dishes, in phenol red-free medium (Invitrogen). FRAP experiments were conducted on a Leica TCS SP5 microscope equipped with a 63 × 1.4 numerical aperture oil-immersion lens. During experiments, cells were maintained at 37 °C and 5% CO_2_. A defined circular region of 2 μm diameter (Region of Interest 1 — ROI1) was placed to the nuclear midpoint of cells. GFP was excited using the 488 nm Argon laser line. Fifty pre-bleach images were acquired with 2% of the 488 nm line at 60% Argon laser intensity, followed by double bleach pulses on ROI1 of 0.066 s using the 476 nm and 488 nm laser lines, combined at maximum power. In this manner, at least 60% of the fluorescence in ROI1 was successfully bleached. Following bleaching, 300 images were recorded at 0.066 s intervals. Mean fluorescence intensities of the ROI1, the whole nucleus (ROI2) and an area outside the nucleus for background correction (ROI3) were quantified and exported as comma-separated values. Quantitative analysis of the experimental recovery curves was performed using easyFRAP [29] and model-based analysis was performed using the parameter inference method described previously [30].

### 2.8 Statistical analysis

Statistical differences between two groups of data were assessed using the unpaired *t*-test or the 1-way ANOVA test (for FRAP analysis results) in the GraphPad Prism version 5.04 software; *P* < 0.05 was considered to be significant (*P < 0.05; **P < 0.01; ***P < 0.001).

## 3 Results

### 3.1 Detection and relative quantification of HIF-1α/ARNT complex formation using *in situ* PLA in HeLa cells

**Fig. 1:**
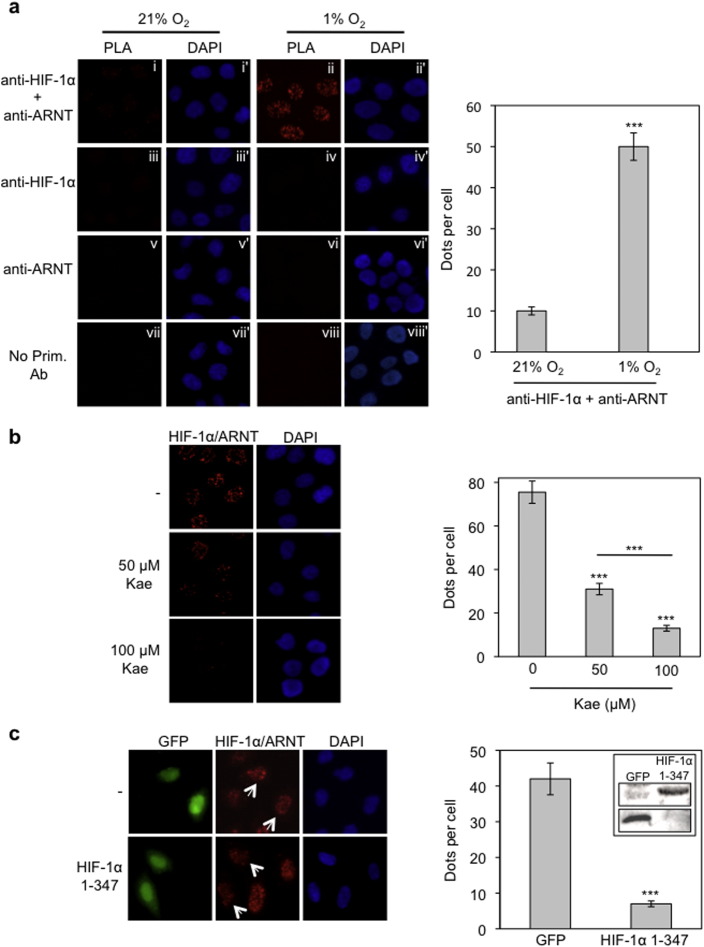
HIF-1α/ARNT complexes can be specifically detected and quantified by *in situ* PLA in HeLa cells. (a) Cells were incubated at normoxia or hypoxia (1% O_2_) for 4 h and processed for the detection of HIF-1α/ARNT interaction using simultaneous incubation with both primary anti-HIF-1 and anti-ARNT antibodies and the *in situ* PLA method (panels i and ii). Treatment of cells with a single primary antibody (panels iii–vi) or no primary antibodies (panels vii and viii) was used as negative controls. Panels i–viii show microscopic images of the PLA signal as nuclear dots while the corresponding panels i′–viii′ show the cell nuclei stained with DAPI. (b) Detection of HIF-1α/ARNT complexes by *in situ* PLA in HeLa cells incubated under hypoxia (1% Ο_2_) for 4 h in the absence or presence of kaempferol (50–100 μΜ). (c) Twenty hours post-transfection, HeLa cells expressing GFP or GFP-HIF-1α 1-347 were incubated for 4 h in hypoxia (1% O_2_) and HIF-1α/ARNT complexes were detected by *in situ* PLA. Arrows point to transfected cells. *Left panels*: microscopical images. *Right panels:* quantification of results presenting the average number of nuclear dots per cell ± SEM (*n* = 50). *Inset in (c)*: immunobloting analysis of cell lysates with an anti-GFP antibody to show expression of GFP alone or GFP-HIF-1α 1-347.

In order to study the regulation of complex formation of endogenous HIF-1 in intact cells, we applied the *in situ* proximity ligation assay (PLA). Using this method, the HIF-1α/ARNT interaction was monitored in HeLa cells that were incubated under normoxic or hypoxic conditions. Following treatment with both anti-HIF-1α and anti-ARNT primary antibodies and analysis by *in situ* PLA, a very weak signal could be detected in cells incubated under normoxia while, in contrast, the signal was drastically amplified in cells grown under hypoxic conditions (Fig. 1a, panels i and ii and chart). The detected signals were specific for the HIF-1α/ARNT complex as no signal was obtained when one or both of the primary antibodies were omitted (Fig. 1a, panels iii–viii). We, therefore, concluded that *in situ* PLA could be used for specific detection of HIF-1α/ARNT heterodimerization in intact cells.

To validate *in situ* PLA as a means to determine the extent of HIF-1α/ARNT complex formation we used two different approaches. Kaempferol, a dietary flavonoid causes mislocalization of HIF-1α to the cytoplasm by inactivation of ERK1/2 [31]. Thus, treatment with kaempferol (50–100 μΜ) under hypoxic conditions is expected to lead to decreased numbers of active HIF-1 complexes inside the nucleus. Indeed, as shown in Fig. 1b, treatment of HeLa cells with kaempferol resulted in a significant and concentration-dependent decrease of nuclear PLA signals. In the second approach, we attempted to disrupt formation of HIF-1α/ARNT complexes when HIF-1α and ARNT proteins are both simultaneously present inside the nucleus. To this end, we used the N-terminal part of HIF-1α, containing its heterodimerization domain that is known to compete with endogenous HIF-1α for its interaction with ARNT [32]. Overexpression of this domain (HIF-1α1-347) as a GFP-tagged protein in HeLa cells led to a strong decrease in the number of PLA signals compared with cells expressing GFP alone (Fig. 1c; white arrows). Taken together, our results show that *in situ* PLA can be reliably used not only to detect but also to quantify the relative extent of HIF-1α/ARNT interaction inside the nucleus.

### 3.2 Overexpression of CK1δ impairs and inhibition of CK1δ stimulates HIF-1α/ARNT complex formation under hypoxic conditions

**Fig. 2:**
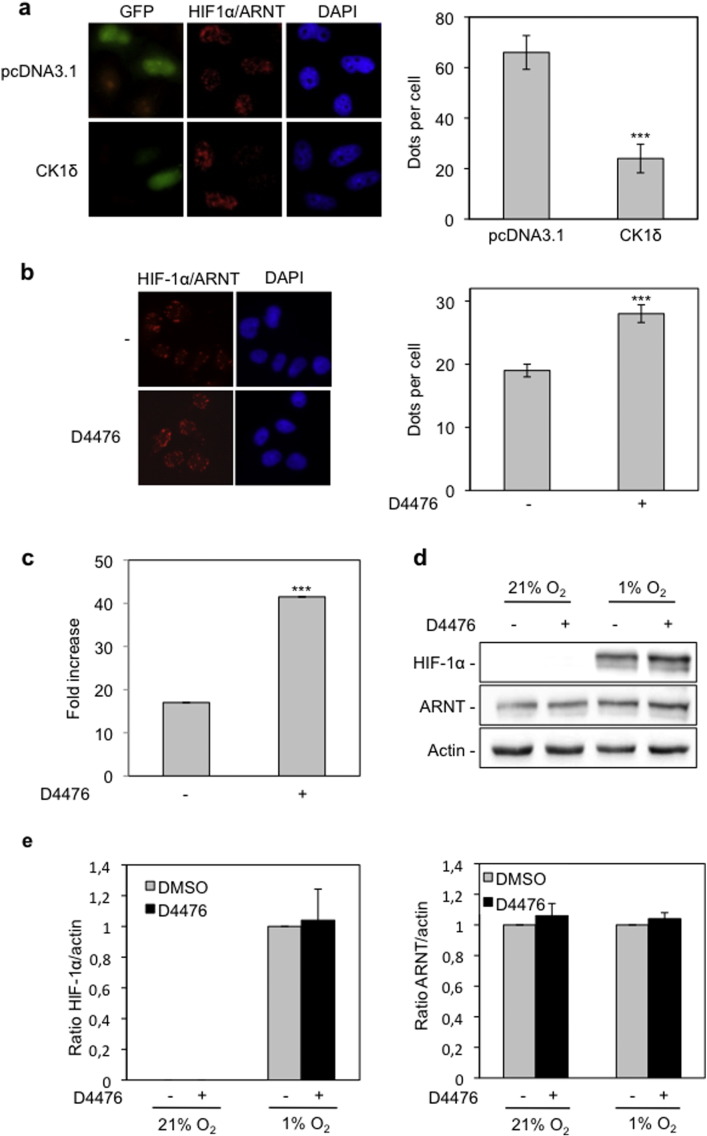
Overexpression of CK1δ impairs and inhibition of CK1δ by D4476 increases formation of HIF-1α/ARNT complexes. (a) HeLa cells were co-transfected with pcDNA3.1 or pcDNA3.1-CK1δ and pEGFP plasmids. Twenty hours post-transfection cells were incubated under hypoxia (1% O_2_) for 4 h and HIF-1α/ARNT complexes were detected by *in situ* PLA. (b) Detection of HIF-1α/ARNT complexes, in the absence or presence of D4476 (10 μΜ) under hypoxia (1% O_2_) for 4 h, by *in situ* PLA. For (a) and (b): *left panels*: microscopical images. *Right panels:* quantification of results presenting the average number of nuclear dots per cell ± SEM (*n* = 50). (c) Determination of HIF-1 transcriptional activity in HeLa cells incubated for 16 h under normoxia or hypoxia (1% O_2_) in the absence or presence of D4476 (10 μΜ). Results are shown as fold increase in relation to the corresponding normoxic conditions and represent the mean of three independent experiments performed in triplicate ± SEM. (d) Western blot analysis of HeLa cells incubated for 4 h under normoxia or hypoxia (1% O_2_) in the absence or presence of D4476 (10 μΜ), for detection of HIF-1α and ARNT protein levels. (e) Histograms show the HIF-1α/actin (left) or ARNT/actin (right) protein levels ratio according to quantification of blots from three independent experiments performed as in (d).

Having established the suitability of *in situ* PLA for generating quantifiable data in a cell-based system, we investigated the effect of CK1δ on the formation of HIF-1 complexes. HeLa cells were co-transfected with a CK1δ overexpressing plasmid (pcDNA3.1-CK1δ) or the corresponding empty vector (pcDNA3.1) and a GFP expressing plasmid (pEGFP; to detect transfected cells) and incubated under hypoxic conditions (Supplementary Fig. 1a). Visualization and quantification of the PLA signals show a significant decrease in the number of HIF-1α/ARNT complexes specifically in cells that overexpress CK1δ compared to control cells that are transfected with the empty vector (Fig. 2a) Therefore, CK1δ does indeed inhibit HIF-1 heterodimerization in intact cells in accordance with our previous *in vitro* experiments [19].

To confirm the negative effect of CK1δ on HIF-1 complex assembly, we used a potent and specific (at 10 μΜ) CK1δ inhibitor, D4476, which has recently become commercially available and used successfully for targeting CK1δ [33–35]. Treatment with the D4476 (10 μΜ), resulted in a significant increase in the number of nuclear HIF-1α/ARNT complexes, as shown by *in situ* PLA in HeLa cells incubated under hypoxia (Fig. 2b). In the presence of D4476, transcriptional activity of endogenous HIF-1 was also drastically enhanced in HeLa cells grown under hypoxia (Fig. 2c). Interestingly, analysis by western blotting confirmed that the stimulation of HIF-1 transcriptional activity by D4476 was not due to an effect on HIF-1α or ARNT expression levels, which remained constant both in the presence and absence of the inhibitor (Fig. 2d and e). Taken together, our data verify that CK1δ inhibits HIF-1 activity by impairing the formation of HIF-1α/ARNT heterodimers.

### 3.3 Phospho-site mutation S247A and inhibition of CK1δ by D4476 both reduce nuclear mobility of HIF-1α in living cells

**Fig. 3:**
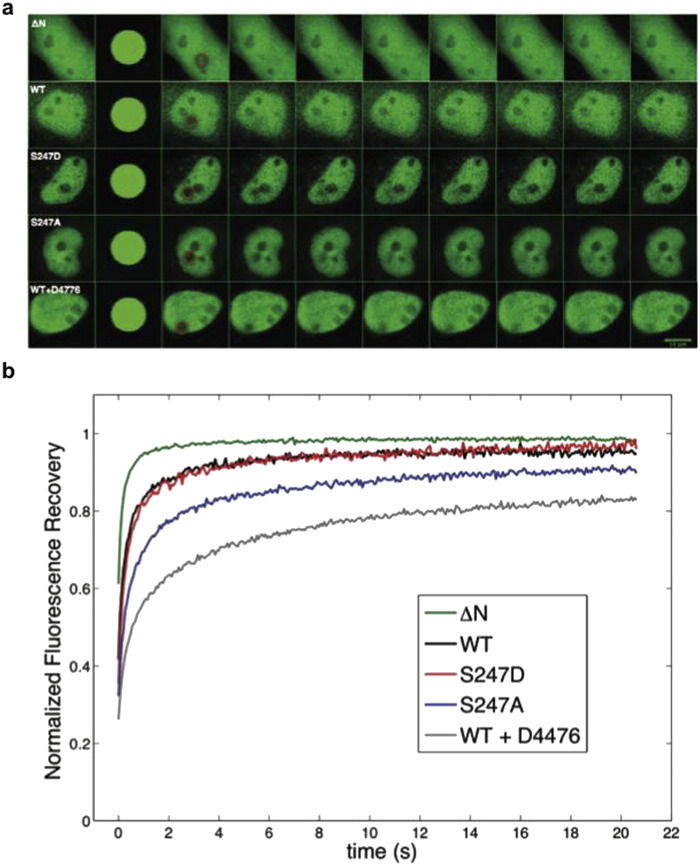
Phospho-site mutation S247A and inhibition of CK1δ by D4476 reduce nuclear mobility of HIF-1α in living cells. (a) Transfected HeLa cells overexpressing the indicating HIF-1α forms tagged with GFP were processed for FRAP analysis 24 h post-transfection. Representative time-lapse images of cells are shown for each GFP-tagged protein. Circles indicate the bleached region. (b) Analysis of FRAP recoveries. Curves represent the mean corrected fluorescence intensities over time for GFP-tagged HIF-1α-ΔΝ, wt HIF-1α, HIF-1α S247A, HIF-1α S247D and wt HIF-1α in the presence of D4476, as indicated.

To investigate the effect of CK1-mediated phosphorylation on HIF-1 in living cells, we applied fluorescence recovery after photobleaching (FRAP) for the determination of HIF-1α intranuclear mobility and kinetics that reflect its ability to form heterodimers that bind stably to chromatin. To this end, we used HeLa cells ectopically expressing GFP-HIF-1α or its mutant forms that abolish or mimic its CK1δ-dependent phosphorylation (Supplementary Fig. 1b). Mutation of Ser247 to alanine (S247A) has been shown to increase the affinity of HIF-1α for ARNT in *in vitro* binding assays, whereas, the phosphomimetic mutation of the same site to aspartate (S247D) has been shown to exhibit the opposite effect [19]. As negative control, we used HeLa cells expressing a GFP-tagged fragment of HIF-1α that lacks the heterodimerization domain (HIF-1α-ΔΝ) and has, therefore, no ability to form heterodimers. FRAP was performed 24 h post-transfection by bleaching a circular area within the nucleus and then monitoring the recovery of fluorescence in the bleached region over time (Fig. 3a, circles). A visual, qualitative examination of the resulting FRAP recovery curves (Fig. 3b and Supplementary Fig. 2) revealed distinct dynamics for the GFP-HIF-1α constructs under normoxia. GFP-HIF-1α-ΔΝ is characterized by rapid and full recovery of fluorescence, indicating a purely diffusive behavior. Wild-type HIF-1α and its phosphomimetic mutant (S247D) are characterized by similar dynamics, whereas S247A, which is unable to be phosphorylated by CK1δ, exhibits decreased recovery. Finally, recovery was further decreased when cells expressing wild type GFP-HIF-1α were treated with the CK1δ inhibitor D4476.

Quantitative analysis of the experimental recovery curves was performed using easyFRAP [29], which allowed the extraction of values for mobile fraction and half-maximal recovery time (t_1/2_) and model-based analysis [30], which permitted determination of the underlying kinetic parameters, namely diffusion coefficient (associated with the speed of diffusion of free molecules), bound fraction (defined as the fraction of molecules that are bound at any given time) and residence time (defined as the time a molecule spends on average in the bound state). These values (Table 1) confirmed the visual observations. More specifically, GFP-HIF-1α-ΔΝ was highly mobile and exhibited a small t_1/2_, significantly faster diffusion coefficient and negligible bound fraction and residence time. These results are consistent with the inability of GFP-HIF-1α-ΔΝ to interact with ARNT and bind to chromatin. Diffusion coefficient values were not significantly different (*P* > 0.05) between all forms of full-length GFP-HIF-1α. Wild-type GFP-HIF-1α and its phosphomimetic mutant S247D were characterized by similar values for t_1/2_, mobile fraction and bound fraction (no statistically significant differences were observed, *P* > 0.05 in all cases), indicating quantitative phosphorylation of GFP-HIF-1α by CK1δ in living cells. In contrast, the phosphodeficient S247A mutant exhibited significantly higher residence time than the mutant S247D (*P* < 0.01), indicative of higher affinity for its heterodimerization partner and stronger binding to chromatin components. In cells treated with D4476 and for wild-type GFP-HIF-1α, recovery was slower (*P* < 0.05 compared to wild-type in absence of D4476; *P* < 0.01 compared to S247D), mobile fraction was lower (*P* < 0.05 compared to either wild-type minus D4476 or S247D), bound fraction was higher (*P* < 0.01 compared to wild-type minus D4476) and residence time was longer (*P* < 0.01 compared to S247D). The differences between mutant GFP-HIF-1α-S247A and wild-type GFP-HIF-1α plus D4476, although they did not reach statistical significance, indicate that modification of HIF-1α Ser247 may not be the sole involvement of CK1δ in HIF-1 regulation.

### 3.4 Inhibition of CK1δ-dependent phosphorylation of HIF-1α facilitates metabolic adaptation of cells to hypoxia

**Fig. 4:**
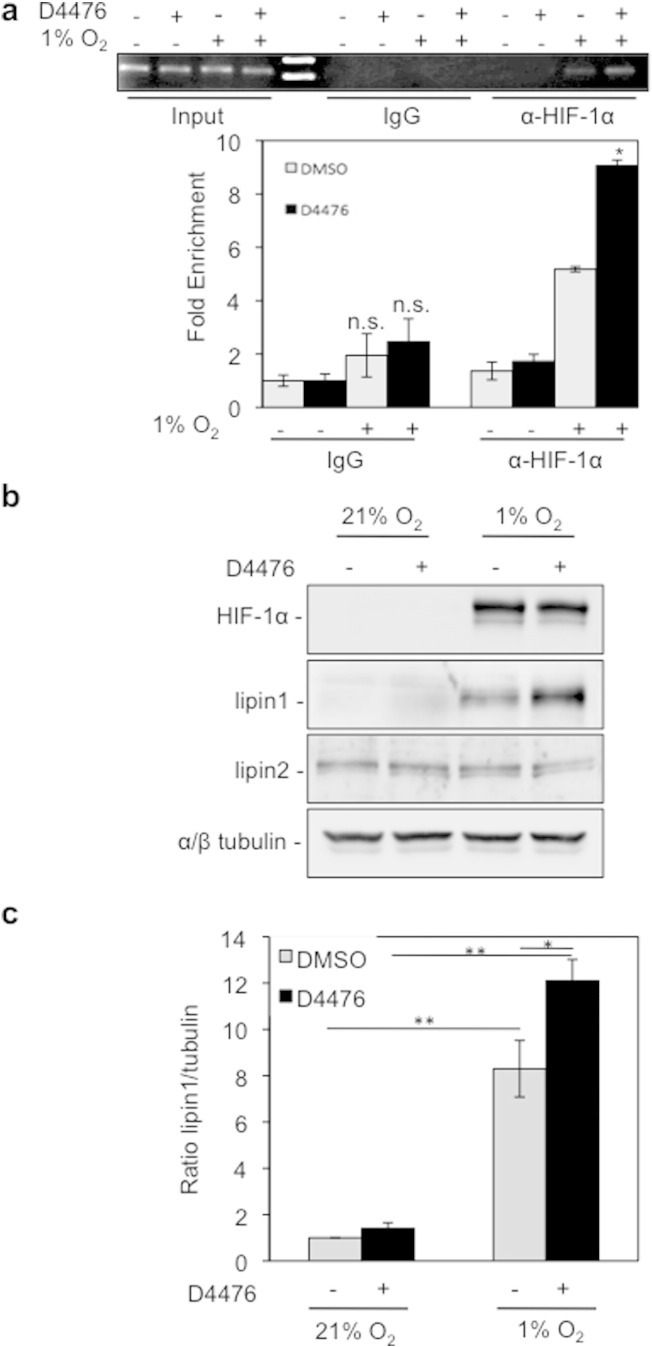
CK1δ inhibition stimulates lipin-1 expression under hypoxic conditions. (a) D4476 increases the interaction of HIF-1α with lipin1 promoter. *Upper:* gel electrophoresis of PCR products amplified from anti-HIF-1α or rabbit IgG chromatin immunoprecipitates of Huh7 cells, incubated for 8 h under normoxia or hypoxia (1% O_2_) in the absence or presence of D4476 (10 μΜ). *Bottom:* quantification of real-time PCR results. Data represent the mean (± SEM) of two independent experiments performed in triplicate. (b) Western blotting analysis of HeLa cells incubated for 24 h under normoxia or hypoxia (1% O_2_) in the absence or presence of D4476 (10 μΜ), for detection of HIF-1α and lipin protein levels. Tubulin was used as loading control. (c) Histogram shows the lipin-1/tubulin protein levels ratio according to quantification of blots from three independent experiments performed as in (b).

To evaluate whether the enhanced HIF-1 heterodimerization caused by CK1δ inhibition is productive in terms of stimulated transcription of HIF-1 target genes, we investigated binding of HIF-1 to the HRE in the promoter of *Lpin1*, a recently identified hypoxia and HIF-1 regulated gene, the product of which, lipin-1, has phosphatidic acid phosphatase activity and is required for up-regulation of triglyceride synthesis and lipid droplet formation under hypoxia [6]. This was first done by chromatin immunoprecipitation using Huh7 cells, in which induction of the *Lpin1* gene by hypoxia is more pronounced [6]. As anticipated, the *Lpin1* promoter region was enriched in anti-HIF-1α immunoprecipitates from hypoxically treated cells in comparison to rabbit IgG immunoprecipitates or anti-HIF-1α immunoprecipitates from normoxic cells. Furthermore, isolation of the promoter was significantly enhanced when D4476 was present (Fig. 4a). Up-regulation of lipin-1 synthesis by CK1 inhibition was further confirmed by analysis of lipin-1 expression levels in HeLa cells grown under the same conditions. As shown in Fig. 4b and c, lipin-1 expression was significantly increased by D4476 treatment under hypoxia, while HIF-1α protein levels remained unaffected. At the same time, expression of lipin-2, which is not an HIF-1 target, remained unchanged (Fig. 4b). We, therefore, conclude that HIF-1α association with the *Lpin1* promoter is stimulated when CK1δ is inhibited, thereby enhancing lipin-1 synthesis.

**Fig. 5:**
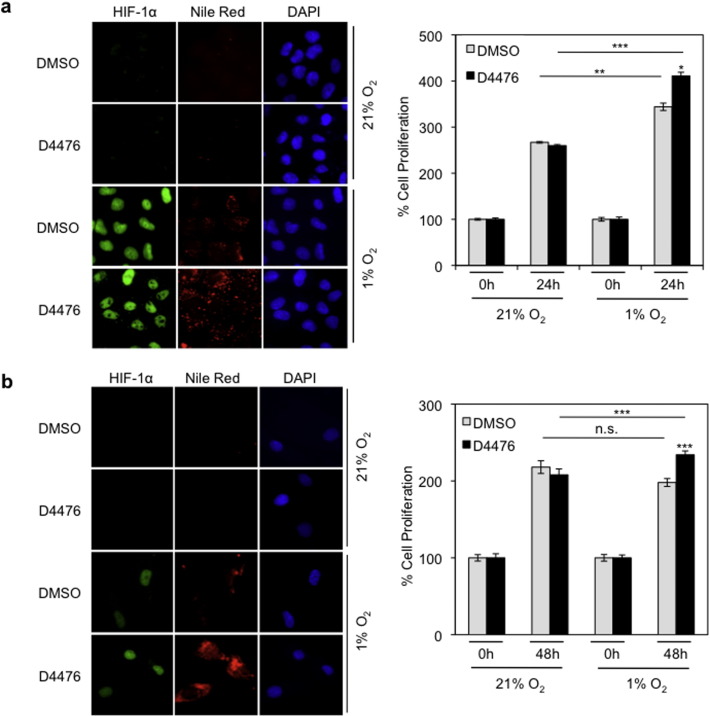
CK1δ inhibition increases lipid accumulation and cell proliferation under hypoxic conditions. Treatment with D4476 (10 μΜ) of HeLa (a) or hSMB (b) cells kept under normoxia or hypoxia (1% O_2_). *Left*: fluorescence microscope images of cells incubated under normoxia or hypoxia (1% O_2_) for 24 h in the absence or presence of D4476 (10 μΜ) and stained with Nile Red to visualize lipid droplets. *Right*: digitized graph of cell proliferation under normoxia or hypoxia (1% O_2_) in the absence or presence of D4476 (10 μΜ) after 24 h (a) or 48 h (b) treatment. Data represent the mean of three independent experiments performed in triplicate and expressed, as percent of the initial number of cells at time zero ± SEM.

To investigate the effect of these changes on triglyceride metabolism we examined the formation of lipid droplets in HeLa cells incubated with D4476 for 24 h under normoxic or hypoxic conditions. Treatment with D4476 significantly increased lipid droplet formation under hypoxia (Fig. 5a, left panel and Supplementary Fig. 3a), indicating increased triglyceride production via up-regulation of the HIF-1 and lipin-1. To see if the changes triggered by CK1δ inhibition would affect cellular adaptation to hypoxia, we measured the proliferation of HeLa under the same conditions. Inhibition of CK1δ did not significantly affect cellular growth rate under normoxic conditions. However, under hypoxia, D4476 caused a small but statistically significant increase in cell proliferation, in agreement with its positive effect on HIF-1 activity (Fig. 5a, right panel). We then tested the effects of CK1δ inhibition on lipid droplet accumulation and cell proliferation in normal, non-cancer cells by using primary, non-transformed human bronchial smooth muscle (hBSM) cells. The results were similar as with HeLa cells (Fig. 5b and Supplementary Fig. 3b), suggesting that the CK1δ-HIF-1α-lipin-1 regulatory axis operates irrespective of the cellular transformation status.

**Fig. 6:**
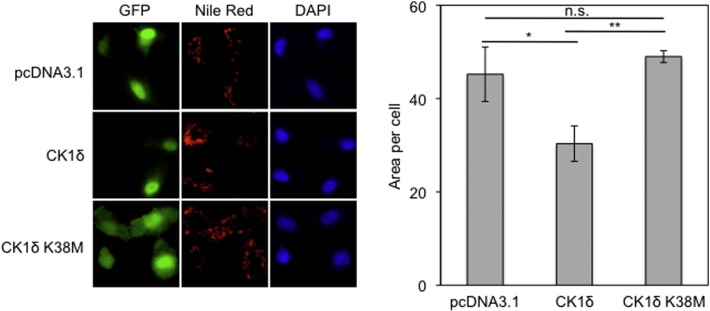
Overexpression of active CK1δ impairs lipid accumulation under hypoxic conditions. *Left*: HeLa cells were co-transfected with pcDNA3.1 or pcDNA3.1-CK1δ wt or pcDNA3.1-CK1δ K38M and pEGFP plasmids. Twenty four hours post-transfection cells were incubated for 24 h under hypoxia (1% O_2_), stained with Nile Red to visualize lipid droplets and observed by fluorescence microscope. *Right:* quantification was performed using ImageJ software and represent the mean area ± SEM of Nile Red staining of 50 cells.

To confirm the loss-of-function experiments using CK1δ inhibition in a positive way, we then examined lipid droplet formation in HeLa cells overexpressing catalytically active CK1δ or, as negative control, the catalytically inactive CK1δ K38M mutant (Supplementary Fig. 1a) [23]. Under hypoxia, lipid droplet formation was decreased when the wild type form of CK1δ was overexpressed, whereas expression of its mutant inactive form K38M did not have any effect on lipid droplet formation (Fig. 6). We conclude that CK1δ limits neutral lipid synthesis under hypoxia, and this is most likely mediated by inhibition of heterodimerization and transcriptional activity of HIF-1.

### 3.5 Stimulation of cell proliferation under hypoxia by CK1δ inhibition is HIF-1- and lipin-1-dependent

The finding that inhibition of CK1δ stimulates cell proliferation under hypoxia is novel and unanticipated. Our data so far suggest that this is the result of increased HIF-1 and lipin-1 activity, triggered by CK1δ inhibition, but we cannot exclude the possibility that cellular proliferation is affected by an unrelated CK1δ target. To address this issue, the involvement of HIF-1 and lipin-1 in adaptation of cells to a hypoxic environment was tested by siRNA-mediated repression of HIF-1α or lipin-1 expression in HeLa cells, kept under normoxia or hypoxia and treated with D4476.

**Fig. 7:**
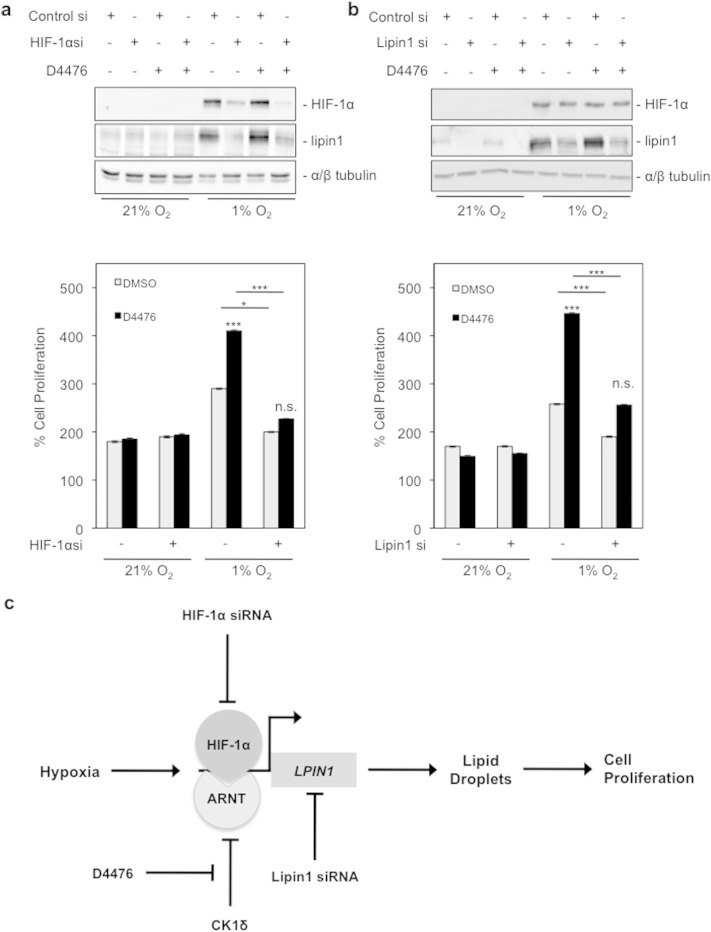
Stimulation of cancer cell proliferation by CΚ1δ inhibition is HIF-1α- and lipin-1-dependent. (a and b) *Upper panels*: results of western blotting analysis for HIF-1α and lipin-1 protein levels after HIF-1α or lipin-1 silencing, respectively. HeLa cells were transfected with control siRNA or siRNA against HIF-1α or lipin-1, and 24 h post-transfection, incubated for 24 h under normoxia or hypoxia (1% O_2_) in the absence or presence of D4476 (10 μΜ). *Bottom panels*: digitized graph of HeLa cell proliferation, treated in the same conditions as described above. Data represent the mean of three independent experiments performed in triplicate and expressed, as percent of the initial number of cells at time zero ± SEM. (c) Schematic model of the mechanism by which CK1δ impairs cell proliferation under hypoxia.

In agreement with our previously published data [6], the siRNA against HIF-1α was effective in reducing the expression of both HIF-1α and lipin-1 under hypoxia (Fig. 7a, upper panel), while the siRNA against lipin-1 decreased the expression of lipin-1 under all conditions (Fig. 7b, upper panel). As expected, knocking down HIF-1α did not affect cell proliferation under normoxia, irrespective of D4476 treatment. However, hypoxic stimulation of proliferation and its further enhancement by D4477 were both greatly abolished when HIF-1α expression was silenced (Fig. 7a, bottom panel). Depletion of lipin-1 did not significantly alter cellular proliferation under normoxia. In contrast, suppression of lipin-1 expression significantly decreased hypoxia-stimulated cellular proliferation, and it almost completely neutralized the positive effect of CK1δ inhibition (Fig. 7b, lower panel). These data lead to the conclusion that increased proliferation of cells under hypoxia requires HIF-1 and also, surprisingly, a lipin-1-mediated function such as, possibly, up-regulation of lipid droplet formation. CK1δ restricts this phenomenon and can limit cellular proliferation under hypoxia by modifying HIF-1α and impairing its association with ARNT and DNA (Fig. 7c).

## 4 Discussion

As HIF-1 is associated with severe pathological conditions such as tissue ischemia and cancer, it is vital to identify in detail the mechanisms that affect its activity in order to develop novel therapeutic approaches. In this study, we documented the negative effect of CK1δ on HIF-1α/ARNT complex formation as well as HIF-1 DNA binding and transcriptional activity, and how this affects metabolic adaptation and proliferation of cells that grow under hypoxia. More specifically, we have shown that CK1δ, by inhibiting HIF-1, impairs lipin-1 expression and lipid droplet formation, processes that are essential for maintaining a high proliferation rate under hypoxia. Therefore, CK1δ appears to have an anti-proliferative role under hypoxia, which may promote cellular homeostasis as well as protect from cancer progression.

It has long been established that HIF-1 is heavily implicated in the shift of energy metabolism in hypoxic cancer cells from mitochondrial oxidative phosphorylation to glycolysis [36]. However, the effects of hypoxia and HIF-1 on lipid metabolism have only recently been studied. Increased lipid droplet accumulation observed under hypoxia can be explained by enhanced de novo fatty acid synthesis, using the acetyl-CoA, overproduced by the combination of glycolytic shift and mitochondrial dysfunction [37,38], and fatty acid synthase (FASN), an essential lipogenic enzyme overexpressed under hypoxia and strongly correlated with cancer progression [39]. Furthermore, recent studies signify the uptake of fatty acids from the growth medium as an important source of lipids in cancer cells under hypoxia [40]. Fatty acid accumulation can be further enhanced by suppression of fatty acid β-oxidation that occurs under hypoxia [5,41]. Thus, hypoxic cancer cells have to deal with the excessive accumulation of fatty acids that can cause lipotoxicity and cell death [42]. According to our model (Fig. 7c), lipotoxicity is avoided by storing fatty acids as neutral triglycerides in lipid droplets via HIF-1-mediated up-regulation of lipin-1 synthesis.

However, the requirement of lipin-1 for the increased proliferation of cancer cells under hypoxia may not only be attributed to the formation of lipid droplets, since diacylglycerol (DAG), the direct product of lipin-1 enzymatic activity, not only is the source of triglycerides but it can also be used for the synthesis of the most abundant phospholipids, phosphatidylethanolamine (PE) and phosphatidylcholine (PC), which participate in membrane biogenesis [43]. Moreover, phosphatidic acid (PA) and DAG, substrate and product, respectively, of lipin-1, play important roles in essential signaling pathways, such as those of mTOR and PKC, and their relative intracellular levels, which are largely determined by lipin activity, may also affect cancer cell proliferation [44,45]. Finally, lipin-1 interacts with and/or modulates the activity of several transcription factors, including members of the peroxisome proliferator-activated receptor (PPAR) family and SREBP, that control the expression of genes involved in lipid metabolism [46,47]. Whatever the case may be, inhibition of HIF-1-dependent lipin-1 expression by CK1δ, appears to restrict cancer cell growth under hypoxia.

Although CK1 isoforms have been implicated in numerous biological functions and also linked to pathological conditions [20], their involvement in cancer cell proliferation and tumor formation is controversial. The role of CK1 in the regulation of pathways associated with cell proliferation, such as those involving p53 or Wnt, can have positive or negative effects depending on cell type and conditions [48–50]. Our previous [19] and current work identifies metabolic adaptation to hypoxia as a new target of CK1, which, in this case, plays an anti-proliferative role by impairing the formation of an active HIF-1 heterodimer. This is in agreement with previous studies examining the effect of CK1 isoforms on the activity of the p53 tumor suppressor protein. Upon cell stress, CK1δ/ε phosphorylates p53 in its N-terminal region, weakens the interaction with MDM2 and, therefore, stabilizes and activates p53 function [51]. Along the same line, reduced expression of CK1α/δ/ε isoforms has been linked to more aggressive carcinoma types, and CK1α has been shown to act as a tumor suppressor when p53 is inactivated [48]. On the other hand, there is also evidence for oncogenic functions of CK1 isoforms in certain types of cancer [20]. A problem with these studies is that expression levels of a CK1 isoform do not necessarily correspond to phosphorylation levels of CK1 targets, as CK1 isoforms are themselves subject to post-translational regulation. The complex multi-layer control of CK1 and its ubiquitous nature create an obstacle in understanding the possible connection between CK1 regulation and the physiological response to hypoxia, which require additional extensive studies.

Apart from cancer, where HIF-1 is associated with pathogenesis and poor patient outcome, HIF-1 has also an important and protective role in a wide range of disorders characterized by ischemia and inflammation [52]. Studies in animal models and patient samples have shown that in ischemic tissues that overexpress HIF-1α, the transcription of genes associated with angiogenesis, vascular remodeling and metabolism is activated, thus, having a favorable impact on tissue health and disease outcome [2]. Moreover, HIF-1α stabilization in conditions such as inflammatory bowel disease, pathogen infection, acute lung injury and organ transplantation has been associated with beneficial results. So, in these disorders, the therapeutic efforts are directed towards augmenting HIF-1 activity. Until now, HIF-1 activators tested for treatment comprise PHD inhibitors that promote HIF-1α stabilization [52]. Although the stability of its alpha subunit is an essential step, full HIF-1 activation also relies on down-stream oxygen-independent processes such as dimerization with ARNT. Our results, showing that inhibition of CK1δ enhances the proliferation of primary normal hBSM cells under hypoxic conditions, suggest that stimulation of HIF-1α/ΑRΝΤ complex formation by CK1δ chemical inhibitors may form the basis of novel therapeutic approaches.

## 5 Conclusions

CK1δ plays an important role in the cellular response to hypoxia by controlling HIF-1 complex formation in living cells. Moreover, CK1δ restricts cellular proliferation under hypoxia by limiting HIF-1 activity, reducing induction of lipin-1 and lowering lipid droplet formation. This novel metabolic function of CK1δ and its modulation by chemical agents can facilitate the development of molecular strategies for the diagnosis or treatment of hypoxia-related pathological conditions.

## Conflict of interest

The authors declare no conflicts of interest.

## Author contributions

M.K. performed the experiments and wrote the paper, G.I. provided reagents and technical assistance, N.-N.G. assisted with FRAP experiments and analysis, M.A.R. analyzed FRAP data, U.L. provided reagents, S.S. provided reagents, Z.L. assisted in FRAP analysis, and G.S. and I.M. designed and supervised experiments and wrote the paper.

## Figures and Tables

**Table 1:** Measured and kinetic parameter estimates of FRAP experiments.

	easyFRAP			Model-based analysis		
GFP-HIF-1α construct	*N*	t_1/2_ (s)	Mobile fraction	Diffusion coefficient (μm^2^/s)	Bound fraction	Residence time (s)
Δ*N*	18	0.15 ± 0.07	0.95 ± 0.04	47.1 ± 5.1	0.01 ± 0.01	–
wt	14	0.29 ± 0.10	0.87 ± 0.11	21.6 ± 16	0.09 ± 0.04	19.2 ± 6.3
S247D	9	0.35 ± 0.19	0.93 ± 0.03	12.5 ± 6.9	0.12 ± 0.05	15.4 ± 5.4
S247A	13	0.74 ± 0.68	0.82 ± 0.16	13.6 ± 11.8	0.18 ± 0.13	20.1 ± 6.6
wt + D4476	15	1.87 ± 2.04	0.70 ± 0.21	10.8 ± 11.6	0.28 ± 0.14	20.8 ± 6.6

*EasyFRAP*: values of half-maximal recovery time (t_1/2_) and mobile fraction for different GFP-HIF-1α constructs, processed as described in Materials and methods. *Model-based analysis:* kinetic parameter estimates of FRAP recovery curves for different GFP-HIF-1α constructs. Numerical values correspond to the mean ± standard deviation of different recovery curves corresponding to different cells (*N* represents the number of cells analyzed in each condition).
