# Supplementary material for: CK1δ restrains lipin-1 induction, lipid droplet formation and cell proliferation under hypoxia by reducing HIF-1α/ARNT complex formation
Source: Cell Signal. 2015 Jun;27(6):1129–40. doi: 10.1016/j.cellsig.2015.02.017 (PMC4390155; doi:10.1016/j.cellsig.2015.02.017)
Supplement: Supplementary file 1 — Supplementary figures [file mmc1.docx]

**SUPPLEMENTERY MATERIAL**

**CK1δ impairs metabolic adaptation to hypoxia by inhibiting**

**HIF-1α/ARNT complex formation**

Maria Kourti^1^, Georgia Ikonomou^1,3^, Nikolaos-Nikiforos Giakoumakis^2^, Maria Anna Rapsomaniki^2^, Ulf Landegren^3^, Symeon Siniossoglou^4^, Zoi Lygerou^2^, George Simos^1,5^ and Ilias Mylonis^1,5^

^1^Laboratory of Biochemistry, Faculty of Medicine, University of Thessaly, Larissa, Greece

^2^Laboratory of Biology, School of Medicine, University of Patras, Rio, Patras, Greece

^3^Department of Immunology, Genetics and Pathology, SciLifeLab, Uppsala University, Uppsala, Sweden

^4^Cambridge Institute for Medical Research, University of Cambridge, Wellcome Trust/Medical Research Council Building, Hills Road, Cambridge CB2 0XY, United Kingdom

^5^To whom correspondance should be adressed:

Prof. George Simos,

Tel: ++30 2410 685723

Fax: ++30 2410 685545

e-mail: [simos@med.uth.gr](mailto:simos@med.uth.gr)

and

Dr. Ilias Mylonis,

Tel: ++30 2410 685578

Fax: ++30 2410 685545

e-mail: [mylonis@med.uth.gr](mailto:mylonis@med.uth.gr)

Laboratory of Biochemistry,

Faculty of Medicine,

University of Thessaly,

41500 BIOPOLIS

Larissa, Greece

**Sup. Fig. 1.** Overexpression of different CK1δ and HIF-1α forms in Hela cells.

(**a**) HeLa cells were transfected with pcDNA3.1, pcDNA3.1-CK1δ or pcDNA3.1-CK1δ K38M plasmids. Twenty-four hours post-transfection cells were lysed and lysates were analysed by SDS-PAGE and western blotting with a rabbit polyclonal antibody against CK1δ and a mouse monoclonal antibody against actin. (**b**) Twenty-four hours post-transfection, HeLa cells expressing GFP alone or different GFP-tagged HIF-1α forms (as indicated) were lysed and lysates were analysed by SDS-PAGE and western blotting with a rabbit polyclonal antibody against HIF-1α and a mouse monoclonal antibody against actin.

**Sup. Fig. 2.**

Mean normalized FRAP recovery curves over time for different GFP-HIF-1α constructs. Grey vertical lines represent mean values ± the respective s.d.

**Sup. Fig. 3.** Quantification of Nile Red staining.

HeLa (**a**) and hSMB (**b**) cells were incubated under normoxia or hypoxia (1% O_2_) for 24 hours in the absence or presence of D4476 (10 μΜ) and stained with Nile Red to visualize lipid droplets. Quantification was performed using ImageJ software and represent the mean pixel area ± s.e.m of Nile Red staining of 50 cells.


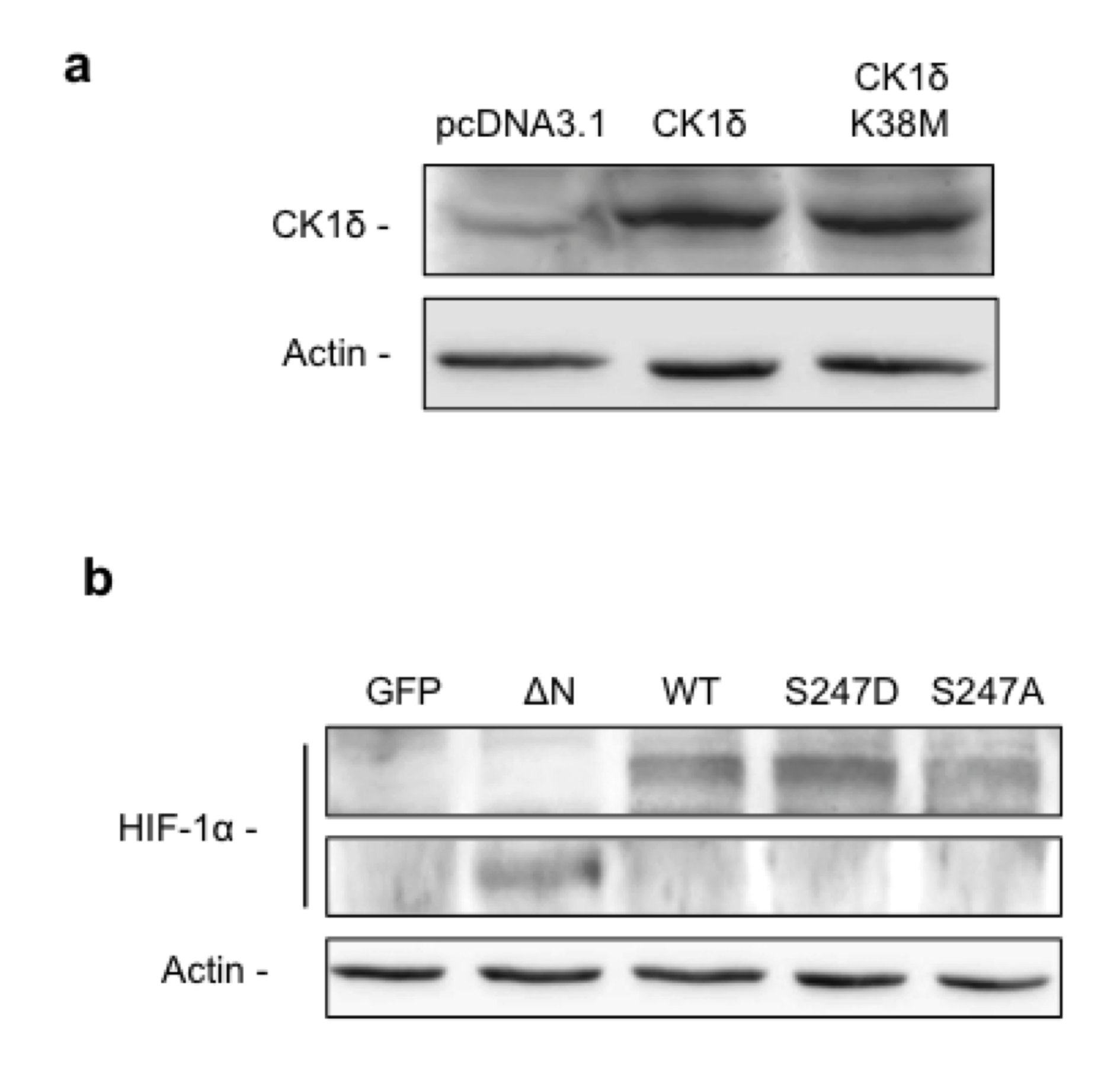


Sup. Fig. 1

Sup. Fig. 2

**
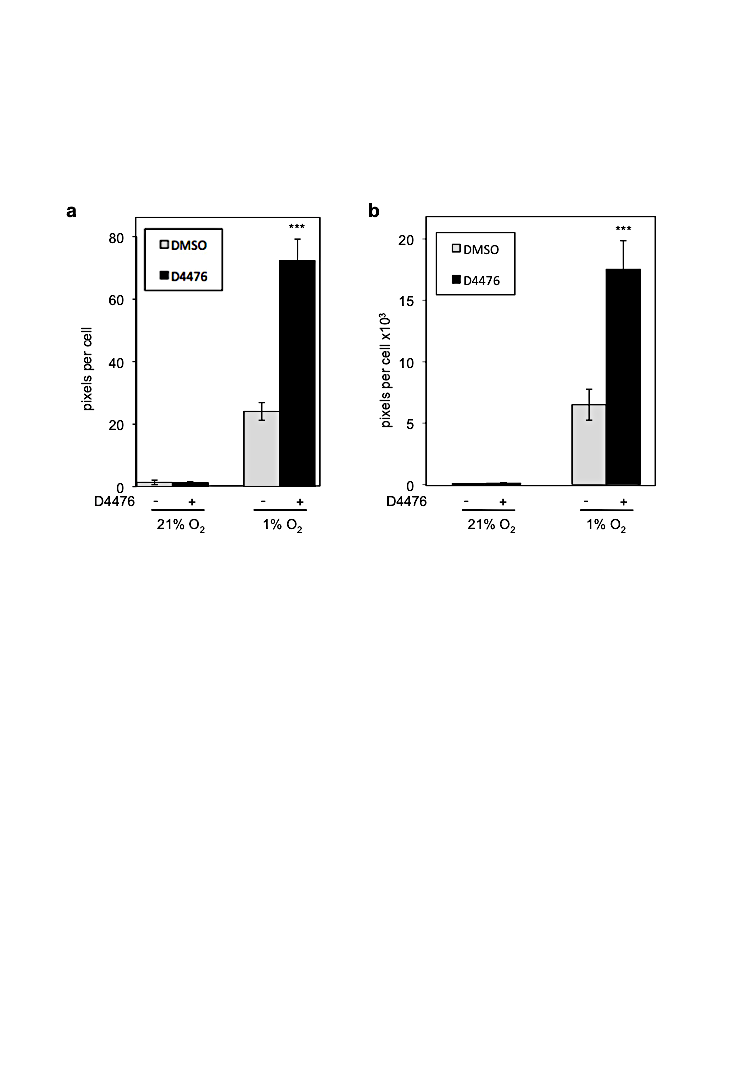
**

Sup. Fig. 3
